# Increased blood neutrophil extracellular traps (NETs) associated with early life stress: translational findings in recent-onset schizophrenia and rodent model

**DOI:** 10.1038/s41398-022-02291-4

**Published:** 2022-12-26

**Authors:** Fabiana Corsi-Zuelli, Ayda Henriques Schneider, Thamyris Santos-Silva, Camila Marcelino Loureiro, Rosana Shuhama, Paulo Rossi Menezes, Francisco Silveira Guimarães, Felipe Villela Gomes, Fernando Queiroz Cunha, Paulo Louzada-Junior, Cristina Marta Del-Ben

**Affiliations:** 1 Department of Neuroscience and Behaviour, Division of Psychiatry, Ribeirão Preto Medical School, University of São Paulo, Ribeirão Preto, São Paulo, Brazil; 2 Center for Research in Inflammatory Diseases – CRID, Ribeirão Preto Medical School, University of São Paulo, Ribeirão Preto, São Paulo, Brazil; 3 Department of Pharmacology, Ribeirão Preto Medical School, University of São Paulo, Ribeirão Preto, São Paulo, Brazil; 4 Population Mental Health Center, University of São Paulo, São Paulo, Brazil; 5 Department of Preventive Medicine, Faculty of Medicine, University of São Paulo, Ribeirão Preto, São Paulo, Brazil; 6 Department of Internal Medicine, Ribeirão Preto Medical School, University of São Paulo, Ribeirão Preto, São Paulo, Brazil

**Keywords:** Biomarkers, Neuroscience, Schizophrenia, Diagnostic markers

## Abstract

Higher levels of interleukin (IL)-6 and elevated neutrophil counts are consistently reported in the blood of patients with schizophrenia. Stressors during childhood and/or adolescence are major socioenvironmental risk factors for schizophrenia and may contribute to immune dysregulation. Previous studies using blood cytokines to stratify patients with schizophrenia suggest that only a subset presents a low-grade inflammatory state. However, these studies have not addressed whether environmental factors such as childhood maltreatment contributed to identifying inflammatory clusters. Moreover, a neutrophil-related mechanism (Neutrophil Extracellular Traps; NETs) central to both the initiation and chronicity of autoimmune and inflammatory diseases has never been investigated in psychiatry. Elevated NETs in schizophrenia may predispose patients to inflammatory and autoimmune diseases resulting in reduced life expectancy. We, therefore, investigated NETs as a novel mechanism and biological target in early schizophrenia and their role together with IL-6 and childhood maltreatment in identifying cluster subgroups. We found increased NETs in the plasma of patients with early schizophrenia (*n* = 78) compared to both their unaffected siblings (*n* = 25) and community controls (*n* = 78), irrespective of sex, body mass index, psychoactive drug use, or tobacco smoking. Increased NETs in patients were unrelated to antipsychotic treatment, which was further tested in vitro using fresh neutrophils. By applying unsupervised two-step clustering analysis, we integrated values of NETs, IL-6, and childhood maltreatment scores. We identified two main clusters; childhood maltreatment scores and NETs were the most important variables contributing to cluster separation (high-CL1 and low-CL2), while IL-6 was the least contributor. Patients allocated in the high-CL1 (61.5%) had significantly higher childhood maltreatment scores, NETs, and IL-6 levels than the remaining groups (patients low-CL2, siblings, and controls high-CL1 and low-CL2). We complemented these findings with a rat model based on stress exposure during adolescence that results in several schizophrenia-like changes in adulthood. We found that adolescent stressed rats had higher NETs and IL-6 levels in serum compared to non-stressed rats with a tendency to produce more NETs from the bone marrow. Altogether, this study brings a novel cellular-based mechanism in schizophrenia that, combined with early-stress, could be useful to identify subgroups for more personalised treatments.

## Introduction

Schizophrenia is one of the most debilitating and chronic psychiatric disorder with substantial variability in the pattern of symptoms and severity [1]. Current antipsychotic treatments are important to control positive symptoms such as delusions and hallucinations, but they still have little benefit on negative and cognitive symptoms [2]. In addition, around one-third of patients do not respond to current antipsychotic drugs [3, 4], and some studies show that about the same proportion of patients displays elevation in blood inflammatory cytokines that may predict poor response to treatment [5, 6]. Therefore, understanding immune mechanisms in schizophrenia may help to tailor better treatments to specific biological subgroups.

Several studies have described immune dysfunction in schizophrenia and psychosis. Large epidemiological data show a positive association between schizophrenia and autoimmune and infectious diseases [7, 8]. Accordingly, meta-analyses consistently show elevated levels of cytokines and acute-phase proteins in the peripheral blood of patients with psychosis compared to healthy controls [9–11]. Interleukin (IL)-6 stands out for being increased both in the cerebrospinal fluid [12] and blood of patients with psychosis, even before antipsychotic initiation [10].

Despite advances in research to understand the relationship between schizophrenia and the immune system, a remarkable gap is the scarcity of investigations based on mechanisms related to peripheral blood immune cells. Immune cell-based research could help to identify components of the immune system most relevant to the persistently reported low-grade inflammation and its impact on related neurochemical and behavioural changes [13, 14]. In schizophrenia, meta-analyses show that total white blood cell counts are significantly elevated in patients relative to controls; a particular increase in neutrophils is observed among drug naïve first-episode psychosis (FEP), acute relapse, and chronic patients, independent of confounding variables such as sex, age, body mass index (BMI), and smoking [15–20].

The rationale for increased neutrophils in schizophrenia and psychosis is unknown, and neutrophil-related mechanisms potentially contributing to low-grade inflammation have never been investigated in the field. In 2004, Brinkmann and colleagues described a new function of neutrophils: the formation of Neutrophil Extracellular Traps (NETs), web-like structures composed of fibres, DNA, histones, and various neutrophil cytosolic proteins, such as myeloperoxidase (MPO) and elastase [21]. The process of NETs formation is called NETosis, and this process is involved in the killing of bacteria, fungi, parasites, and viruses [21–24]. Interestingly, however, NETs have been implicated in various non-infectious diseases such as rheumatoid arthritis, lupus, psoriasis, diabetes, and cardio-metabolic disorders [25–32], some of which are comorbidities for psychosis and schizophrenia [8, 33]. NETs have also been involved in brain disorders, including stroke, traumatic brain injury, multiple sclerosis, and Alzheimer’s disease [34, 35]. Studies have been investigating anti-NETs compounds to treat or prevent clinical inflammatory diseases [27, 29, 32, 36, 37]. However, NETs have never been investigated in psychiatric disorders. The investigation of NETs in psychiatry is timely and could help to uncover novel mechanisms and treatment targets.

Early-life stress, such as childhood maltreatment, can trigger the development of mental illness and immune perturbations in adulthood [38, 39]. However, not all patients with psychosis have been exposed to childhood trauma or show signs of immune dysfunction [40]. These potentially reflect heterogeneity in disease and mechanisms. Previous studies attempting to identify subgroups of patients based on immunological variables such as cytokines suggested that only a subset has a low-grade cytokine profile [41–48]. Nevertheless, these studies have not addressed the role of environmental factors in such immune deregulation profile. This raises the question of whether childhood maltreatment may have contributed to identifying immune-related subgroups in previous studies.

Here we investigated NETs as a novel cellular mechanism in schizophrenia, and its role together with IL-6 and early stress in identifying cluster subgroups. Using both clinical samples and a rodent model, we hypothesised that a) patients with early schizophrenia would have higher NETs and IL-6 compared to both their unaffected siblings and controls; b) higher NETs and IL-6 would occur particularly in identified clusters of patients with a history of childhood maltreatment; and c) rodents exposed to adolescent stress would display higher NETs and IL-6 levels in serum together with higher release of NETs from neutrophils isolated from the bone marrow compared to non-stressed rats.

## Materials and methods

### Participants

We used data available from the *Schizophrenia and Other Psychoses Translational Research: Environment and Molecular Biology* (STREAM) study. STREAM was a three-year (April 2012 – March 2015) incidence and case-sibling-control investigation conducted in the Ribeirão Preto catchment area (São Paulo, Brazil) [49] as part of the international multicentre consortium *European Network of National Schizophrenia Networks Studying Gene-Environment Interactions* (EU-GEI) [50].

In the original STREAM study, a total of 507 participants (166 FEP patients, 76 unaffected siblings of patients, and 265 community-based controls), aged 16–64 y/o and residing in the Ribeirão Preto catchment area, accepted to take part in a cross-sectional study that included blood collection [40]. The current investigation is a subsample of the cross-sectional study and included all the recruited patients with early schizophrenia spectrum (*n* = 78), sex- and age-matched controls (*n* = 78), and available unaffected siblings of patients with early schizophrenia spectrum (*n* = 25).

### Inclusion criteria

Patients were eligible to participate in the STREAM study if they had contacted mental health services due to a first episode of psychosis not originating from substance intoxication or other medical condition. All the patients were relatively stable at the time of assessment.

Community controls were recruited from the same catchment area of patients to ensure the representativeness of the catchment area’s population at risk for psychosis. They were randomly selected following census blocks defined by the Brazilian Official Census Bureau (IBGE, 2010), with sex, age, and economic profile stratification. Controls had no history of psychotic symptoms throughout life.

Unaffected siblings were invited to participate following the patient’s agreement and if they met the criteria of absence of a lifetime history of psychotic symptoms.

### Exclusion criteria

None of the 181 participants was receiving treatment with anti-inflammatory/immunosuppressive drugs at the time of blood collection or during the past month, and none were pregnant, lactating, nor presented with any chronic/acute medical condition that could potentially influence the biological variables of interest (see [40, 51] for details).

### Clinical and sociodemographic assessments

Clinical and sociodemographic assessments were systematically performed by mental health researchers with weekly supervision from the senior staff [49]. For all the participants, formal psychiatry diagnosis was obtained using the Structured Clinical Interview for DSM-IV, clinical version (SCID-CV) [52, 53]. Illness severity was assessed with the Brief Psychiatric Rating Scale (BPRS) [54, 55]. For patients, information on psychosis onset age, pharmacological treatment initiation, and duration of untreated psychosis (DUP), were obtained using the Nottingham Onset Schedule [56]. Sociodemographic data were obtained using the Medical Research Council Sociodemographic Schedule [57]. Information on lifetime use of cannabis and other psychoactive drugs (cocaine/crack, inhalants, sedatives, amphetamine, hallucinogens) was assessed using the Cannabis Experience Questionnaire (CEQmv_EU-GEI_) [58]. Information on tobacco smoking during the last 12 months relative to the interview was obtained from the Composite International Diagnostic Interview [59].

### Childhood maltreatment

A history of childhood maltreatment was investigated using the Childhood Trauma Questionnaire (CTQ) short form [60, 61]. The CTQ is a self-report questionnaire that retrospectively assesses the history of childhood maltreatment. It contains 25 items rated on a 5-point Likert scale (1 = never; 5 = very often true), used to investigate exposure to abuse (emotional, physical, sexual) and neglect (emotional and physical). We used the CTQ total scores, which result from the sum of values of the five subscales and range from 25 to 125 points [40]. We also considered scores of each subtype of childhood maltreatment in exploratory analyses.

### Biological samples

Peripheral blood samples were obtained after diagnostic evaluation and processed accordingly [40, 51]. Plasma samples from all the participants were then used for both cytokine and NETs quantification.

#### NETs in human plasma

NETs were quantified in human plasma using the Quant-iT PicoGreen kit (Thermo Fisher Scientific, USA, CAT: P11496) as detailed in the Supplementary material and previously described [62].

#### Fresh human neutrophils stimulated with antipsychotic drugs in vitro

To evaluate the effect of antipsychotic drugs on human NETs levels, fresh neutrophils were obtained and isolated from the peripheral blood of a healthy donor using Percoll gradients (72%, 63%, 54%, and 45%) [31]. After centrifugation, polymorphonuclear leukocytes accumulated as a band between 72% and 63% Percoll. Total cell numbers were counted using light microscopy. The percentage of neutrophils was determined microscopically through haematoxylin and eosin staining. One million neutrophils per treatment group were incubated in an HBSS medium (Corning, USA) and then stimulated with phorbol myristate acetate (PMA, 50 nM; Sigma-Aldrich, CAT: P8139), which is known to induce the formation of NETs in vitro [63, 64], and/or the first-generation antipsychotic haloperidol (18 and 25 ng/mL; Nortec Química, Brazil, LT: 544220) or the second-generation antipsychotic risperidone (20 and 40 ng/mL; Zhejiang Huahai Pharmaceutical, China, LT: 798021). Antipsychotics concentrations were chosen based on the optimal plasma levels for therapeutic effects [65]. One hour after stimulation with drugs, neutrophils were incubated at 37 °C for 3 h, and the supernatants were used to determine NETs concentration, using the same protocol described in the Supplementary material.

#### IL-6 in human plasma

Twenty-five microliters of plasma were used for cytokine quantification (pg/mL) using the Milliplex MAP human cytokine magnetic bead panel (EDM, Millipore, USA, CAT: HCYTOMAG-60K) and analysed on a Luminex-200 System (Luminex, USA). The results were then reported on the xPOTENT software v3.1 and corrected using the Milliplex Analyst software. The essay was performed on 96-well plates according to the manufacturer’s instructions, as detailed previously [40, 51]. All the analytes had an accuracy (% recovery in serum matrix) greater than or equal to 80%. The intra- and inter-assay coefficients of variation were below 15% and 20%, respectively.

### Rodent model

Juvenile male Sprague-Dawley rats (postnatal day, PND 24) were obtained from the Central Animal Facility of the University of São Paulo, Ribeirão Preto. Rats were allowed to acclimate for one week at the local animal facility (Department of Physiology) before beginning experiments (PND31). Animals were randomly housed (3–4 animals per cage) in a temperature (22 °C) and humidity (47%) controlled environment (12 h light/dark cycle; lights on at 6 AM) with *ad libitum* access to food and water.

Adolescent male rats were exposed to a combination of daily inescapable footshock (from PD31–40) and three restraint stress sessions (PD31, 32, and 40), as described elsewhere [66, 67] and detailed in the Supplementary material. Naïve animals were left undisturbed in their home cages. This stress protocol induces behavioural and electrophysiological abnormalities in late adolescence and adulthood, resembling schizophrenia [66, 67]. Only males were used in this study since we previously found that female rats were resistant to present behavioural and electrophysiological changes after exposure to this stress protocol [68].

All experiments were conducted according to the National Institutes of Health guidelines for the Care and Use of Laboratory Animals and were approved by the Institutional Animal Care and Use Committee of the University of São Paulo, Ribeirão Preto Medical School (#155/2018).

### NETs and IL-6 measurement in serum

Ten days after the stress protocol (PND51), animals were anesthetised with urethane (2.5%, 1 mL/kg, i.p.). A cardiac puncture was performed to collect blood. The blood samples were centrifuged at 16,000 *g* at 4 °C, and the supernatants (serum) were collected and kept at −80 °C for further analysis. NETs and IL-6 levels were measured using an ELISA assay with paired antibodies (R&D Systems, USA; CAT: R6000B), as previously described [69].

### NETs quantification from fresh neutrophils isolated from rats’ bone marrow

We also measured levels of NETs released from fresh neutrophils isolated from rats’ bone marrow. Rat tibias were collected in PBS to access bone marrow cells. These cells were deposited over the gradient of different Percoll concentrations (72% and 65%) to isolate neutrophils. After, neutrophils were lysed, washed, and counted in Neubauer’s chamber (98% of purification). One million neutrophils per animal were plated in PBS + BSA 1% and incubated for 4 h. Supernatants were stocked at −80 °C, and NETs concentrations were quantified from supernatants as previously described.

### Statistical analysis

#### Clinical sample

Demographic and clinical data were analysed using descriptive statistics according to data distribution. BMI data were missing for 26 participants (14.4%). Considering the potential influence of BMI on inflammatory variables and to optimise our sample size, a fully conditional specification imputation model was performed by predictive mean matching using five multiple imputation methods [51].

#### Biological variables

NETs and IL-6 values obtained from human plasma were natural-log transformed to minimise data skewness. Correlations between NETs and IL-6 were tested using Bivariate Pearson’s correlation. Group differences on both measures were evaluated using analysis of variance with Bonferroni *post-hoc*. Group differences were further analysed using general linear models to adjust for sex, BMI, psychoactive drug use, and tobacco smoking. We used Bivariate Pearson’s correlation in the patient group to test correlations between biological and clinical variables (psychosis age of onset, duration of untreated psychosis (DUP), duration of pharmacological treatment, and duration of psychosis). We used a two-way ANOVA with Bonferroni *post-hoc* to test the effect of antipsychotics on NETs in vitro.

#### NETs, IL-6, and childhood maltreatment; subgroups identification

We used unsupervised two-step clustering analyses to identify potential subgroups and account for heterogeneity in measures of NETs, IL-6, and childhood maltreatment in our sample. See details in the Supplementary material. This approach has been used by other studies in the field to account for potential heterogeneity in measured variables [44, 70, 71]. The identified clusters were then tested for differences in biological (unadjusted and adjusted models), sociodemographic, and clinical variables. We also performed exploratory analyses to test the impact of subtypes of childhood maltreatment on cluster separation.

#### Rodent model

Data are presented as mean ± standard deviation (SD). We verified the homogeneity of variances using Bartlett’s test and the data distribution using the Shapiro-Wilk test. To evaluate the effect of adolescent stress on NETs and IL-6 levels, we performed parametric analyses (Student’s *t*-test). The sample size was based on previous studies from our group [66, 72].

All analyses were performed using SPSS v28.0 and GraphPad Prism 8.0. Results were considered significant at *p* ≤ 0.05 (two-sided).

## Results

### Clinical sample

#### Demographic and clinical features

The groups did not differ in age. However, early schizophrenia patients were more often males compared to their siblings, with siblings having the highest proportion of females (68%) compared to both patients and controls (*p* < 0.001). Patients had slightly lower BMI mean than controls but had the highest frequency of tobacco smoking and psychoactive substance use compared to both controls and siblings (*p* < 0.01). CTQ total scores were higher in both early schizophrenia and their siblings compared to controls (*p* < 0.001). Half of the patients were under pharmacological treatment for 13 weeks. Further clinical characteristics are described in Table 1.

**Table 1:** Socio-demographic and clinical variables (*n* = 181).

Variables	Controls	Patients	Siblings	*p*
	(*n* = 78)	(*n* = 78)	(*n* = 25)	
Male, *n* (%)^d^	54 (69.2)	54 (69.2)	8 (32.0)	**0.002**^**a,b**^
Age, mean (SD)^e^	30.3 (11.9)	30.1 (12.3)	29.8 (8.7)	0.881
Body mass index (kg/m^2^), mean (SD)^e^	26.5 (5.8)	24.0 (4.5)	25.8 (5.2)	**0.035**^**c**^
Tobacco smoking (yes), *n* (%)^f^	8 (10.3)	30 (38.5)	5 (20.0)	**<0.001**^**c**^
Lifetime psychoactive substance use (yes), *n* (%)^f^	16 (20.5)	49 (62.8)	5 (20.0)	**<0.001**^**a,c**^
Childhood trauma questionnaire (total score)^e^	38.2 (13.2)	40.7 (14.2)	41.2 (13.9)	**<0.001**^**b,c**^
Psychosis age of onset, mean (SD)	-	28.4 (12.2)	-	-
DUP (weeks), median (min-max)	-	20.5 (0–1292)	-	-
Pharmacological treatment (weeks), median (min-max)	-	13.0 (0–155)	-	-
Duration of psychosis (weeks), median (min-max)	-	53 (4–1394)	-	-
BPRS, mean (SD)^e^	0.45 (1.43)	8.41 (6.44)	0.72 (1.46)	**<0.001**^**a,c**^
Pharmacological treatment				
Antipsychotics (AP)	-	50 (64.1)	-	-
Antidepressants (AD)		1 (1.3)		
AP + AD	-	10 (12.8)	-	-
AP + Mood Stabilisers (MS)	-	6 (7.7)	-	-
AP + AD + MS	-	3 (3.8)	-	-
None	-	8 (10.3)	-	-

*SD* Standard Deviation, *DUP* Duration of Untreated Psychosis, *BPRS* Brief Psychiatric Rating Scale.

Pairwise comparison: ^a^Patients *v*. Siblings; ^b^Siblings *v*. Controls; ^c^Patients *v*. Controls.

^d^Pearson’s Chi-square test, ^e^Kruskal–Wallis, ^f^Fisher’s Exact Test

Significant results are depicted in bold.

#### NETs are elevated in early schizophrenia patients

**Fig. 1:**
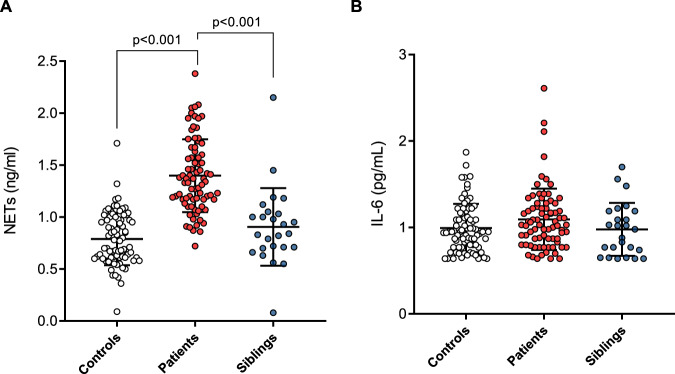
Group differences in the concentration of plasma NETs and IL-6. **A** Plasma NETs (ng/mL) are significantly elevated in early schizophrenia patients (*n* = 78) compared to both their unaffected siblings (*n* = 25) and community controls (*n* = 78). **B** Plasma IL-6 (pg/mL) levels did not differ among the groups. One-way analysis of covariance with Bonferroni post-hoc adjusted for sex, BMI, tobacco smoking, and other psychoactive drug use. NETs and IL-6 (mean, SD) are represented as natural-log transformed values.

Group differences in plasma NETs and IL-6 are described in Fig. 1A, B and Supplementary Table 1. Our unadjusted model showed that early schizophrenia patients had significantly higher NETs levels than their siblings and controls (*p* < 0.001). NETs were higher in patients who were tobacco smokers compared to non-smokers (mean ± SD: 3.80 ± 1.87 *v*. 3.01 ± 1.46, *p* = 0.037). However, the between-group difference remained significant after adjusting for sex, tobacco smoking, psychoactive substance use, and BMI (*p* < 0.001). On the other hand, no group difference was found for IL-6 plasma levels, neither in unadjusted nor in adjusted models (*p* > 0.05).

NETs and IL-6 were not significantly correlated when tested in the whole sample or separately in each group (*p* > 0.05). In patients, neither NETs nor IL-6 levels correlated with the duration of pharmacological treatment or other clinical features (*p* > 0.05 for all). IL-6 had a significant but weak positive correlation with psychosis age of onset (*r* = 0.33, *p* = 0.003).

#### Antipsychotic drugs do not increase NETs from freshly isolated neutrophils in vitro

We observed higher levels of NETs in early schizophrenia compared to controls, irrespective of important covariates. We also noticed that NETs levels in plasma were not correlated with the duration of antipsychotic treatment in patients. However, considering that our patients were minimally treated but not drug-naïve, we performed an in vitro assay using fresh neutrophils from a healthy donor to discard further the potential effect of antipsychotics in elevating the levels of human NETs.

**Fig. 2:**
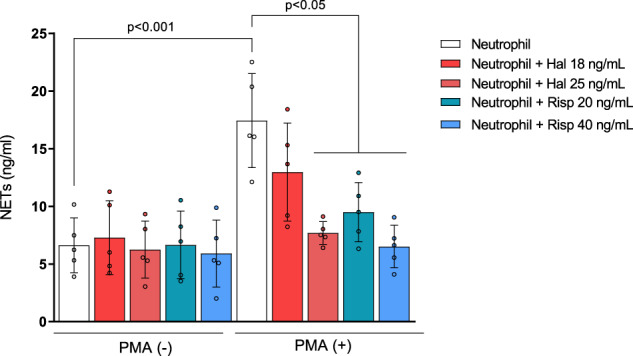
Antipsychotics do not affect NETs levels in the supernatant of fresh human neutrophils in vitro. Haloperidol (Hal; 25 ng/mL) and Risperidone (Risp; 20 and 40 ng/mL) significantly inhibited NETs formation induced by PMA. Two-way analysis of variance followed by Bonferroni *post-hoc*. Data are expressed as mean ± SD.

As expected, incubation of human neutrophils with PMA significantly increased NETs from supernatants compared to unexposed neutrophils (*p* < 0.001). None of the concentrations tested for haloperidol or risperidone induced the formation of NETs. On the contrary, both haloperidol (25 ng/mL) and risperidone (20 or 40 ng/mL) significantly inhibited NETs levels induced by PMA (Fig. 2).

#### Plasma NETs, IL-6, and history of childhood maltreatment; subgroups identification

**Fig. 3:**
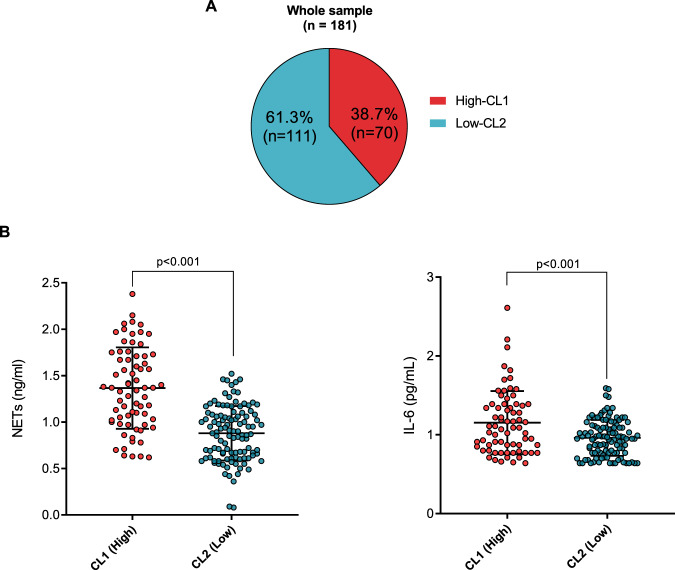
Sample stratification in the whole sample (*n* = 181) using unsupervised two-step cluster analysis and subgroup differences in NETs and IL-6 plasma levels. **A** Two main clusters were identified when integrating values of childhood maltreatment scores, NETs, and IL-6 levels. Cluster 1 (high-CL1) represents participants with higher scores of childhood maltreatment and levels of NETs and IL-6, whereas cluster 2 (low-CL2) represents the opposite. **B** Participants allocated to high-CL1 had significantly higher levels of NETs and IL-6 than the low-CL2 (one-way analysis of covariance with Bonferroni post-hoc adjusted for tobacco smoking and other recreational drugs). NETs and IL-6 (mean, SD) are represented as natural-log transformed values.

Unsupervised two-step clustering analysis using the whole sample (*n* = 181) identified two main clusters (CL1 and CL2) when integrating values of NETs, IL-6, and childhood maltreatment scores. As shown in Fig. 3A, 38.7% (70 out of 181) participants were identified as CL1, while the remaining 61.3% (111 out of 181) were allocated to CL2. CTQ total scores were the most important variable contributing to cluster separation, followed by NETs levels. IL-6 levels were the least contributor (Supplementary Fig. 1).

When testing differences in sociodemographic variables, we observed that clusters did not differ in age, sex, or BMI. However, CL1 differed significantly from CL2 on frequencies of tobacco smoking (CL1: 40%; CL2: 13.5.5%, *p* < 0.001), psychoactive substance use (CL1: 54.3%; CL2: 28.8%, *p* < 0.001), and childhood maltreatment total scores [mean (SD) CL1: 48.6 (15.3); CL2: 31.6 (5.2), *p* < 0.001].

In addition, CL1 had significantly higher NETs and IL-6 in plasma than CL2 (*p* < 0.001 for both), even after adjusting for tobacco smoking or psychoactive substance use (Fig. 3B, Supplementary Table 2). Based on these findings, we named CL1 as “inflamed and early-stressed (high-CL1)” and CL2 as “lower inflamed/stressed (low-CL2)”.

**Fig. 4:**
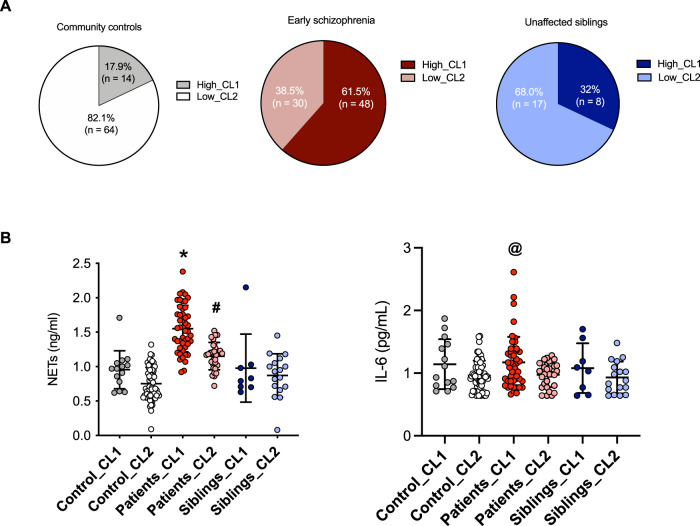
Distribution of clusters among early schizophrenia patients (*n* = 78), their unaffected siblings (*n* = 25), and community controls (*n* = 78), and subgroup differences in NETs and IL-6 plasma levels. **A** High (CL1) and low (CL2) clusters were identified using unsupervised two-step cluster analysis after integrating childhood maltreatment scores, NETs, and IL-6 levels. Cluster 1 (high-CL1) represents participants with higher scores of childhood maltreatment and levels of NETs and IL-6, whereas cluster 2 (low-CL2) represents the opposite. **B** Clusters subgroups differed significantly on levels of NETs and IL-6 (one-way analysis of covariance with Bonferroni post-hoc adjusted for sex, tobacco smoking, and other recreational drugs). *Higher NETs in patients high-CL1 vs all the remaining groups (*p* < 0.001); #Higher levels of NETs in patients low-CL2 vs controls low-CL2 (*p* < 0.001); @Higher levels of IL-6 in patients high-CL1 vs participants in the low-CL2 (patients: *p* = 0.049; siblings: *p* = 0.025; controls: *p* = 0.004). NETs and IL-6 (mean, SD) are represented as natural-log transformed values.

As a next step, we used the preceding cluster separation to perform the same analyses but now considering diagnoses for group stratification. Notably, most of the early schizophrenia patients were identified as belonging to the high-CL1 (*n* = 48, 61.5%), whereas most of the siblings (*n* = 17, 68%) and controls (*n* = 64, 82.1%) pertained to the low-CL2 (Fig. 4A).

We found subgroup-specific differences in the history of childhood maltreatment among the clusters (*p* < 0.001). Patients in the high-CL1 scored significantly higher on CTQ total scores than patients, siblings, and controls in the low-CL2; other differences are described in Supplementary Table 3.

After, we tested if the cluster subgroups differed on biological variables (Fig. 4B, Supplementary Table 4). For NETs, we observed that patients allocated in the high-CL1 had significantly higher levels of NETs than all the remaining groups (patients in CL2 and siblings and controls in both CL1 and CL2; adjusted *p* < 0.001). Furthermore, patients in the lower inflamed/stressed subgroup CL2 had significantly higher NETs levels than controls in the low-CL2 (adjusted *p* < 0.001). For IL-6, we found that patients in the inflamed and early stressed group CL1 had higher levels than participants in the lower-CL2 (patients: *p* = 0.049, siblings: *p* = 0.025, and controls: *p* = 0.004; adjusted values).

Finally, we investigated if the two clusters of patients differed on measured clinical variables, but no difference was found (all *p* > 0.05).

##### Contribution of childhood maltreatment subtypes to cluster separation

Our exploratory analyses testing the impact of subtypes of childhood maltreatment on cluster separation (Supplementary Fig. 2) showed that emotional neglect, physical neglect, and emotional abuse had the strongest impact. Physical and sexual abuse were the least contributors. Stratification based on childhood maltreatment subtypes showed that patients high-CL1 or low-CL2 had higher NETs than the remaining high- or low-clusters of siblings and controls (adjusted *p* < 0.001). No difference among the groups was found for IL-6 in these exploratory analyses (all results are detailed in Supplementary Tables 5-8).

### Rodent model

#### Adolescent stressed rats show increased NETs and IL-6 levels in the serum and increased NETs released from fresh bone-marrow isolated neutrophils

**Fig. 5:**
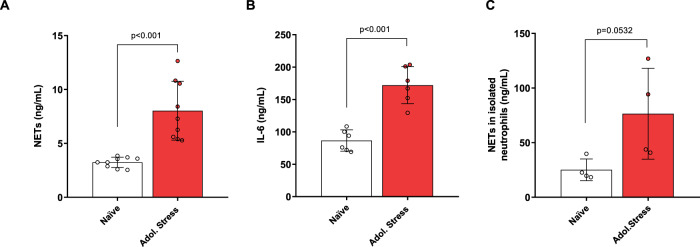
Effect of stress during adolescence on levels of IL-6 and NETs measured in the plasma and bone marrow of rats. **A** NETs (ng/mL) levels are significantly elevated in the serum of adolescent stressed rats. **B** IL-6 levels (ng/mL) are significantly elevated in the serum of adolescent stressed rats. **C** Freshly neutrophils isolated from the bone marrow of adolescent stressed rates tended to produce more NETs than controls. T-test analysis. Data are expressed as mean ± SD.

Adolescent-stressed rats showed increased serum levels of NETs and IL-6 than non-stressed rats (NETs: *t*_16_ = 5.18, *p* < 0.001; IL-6: *t*_10_ = 6.33, *p* < 0.001; Fig. 5A, B). Moreover, fresh neutrophils isolated from stressed rats tended to produce more NETs (*t*_6_ = 2.40, *p* = 0.053; Fig. 5C).

## Discussion

Using a clinical sample of patients with early schizophrenia and a relevant animal model, we showed that: a) neutrophil-related NETs are increased in patients compared to both their unaffected siblings and community controls, irrespective of pharmacological treatment and other confounders; b) a history of childhood maltreatment identified clusters of patients with higher levels of both NETs and IL-6; c) emotional and physical neglect together with emotional abuse were more likely to contribute to cluster separation; d) the effect of early stress was equivalent in rodents; adolescent stressed rats had higher release of NETs and IL-6 in the serum and tended to produce more NETs from the bone marrow compared to non-stressed animals. Altogether, this study brings a novel cellular-based mechanism and strengthens that stress should be considered for identifying immune-related subgroups in schizophrenia.

### NETs are elevated in the plasma of early schizophrenia patients irrespective of confounders

The participation of peripheral cellular immunity, related mechanisms, and their association with low-grade inflammation in schizophrenia is fairly unexplored. Neutrophils are an important part of the innate immune system and the first leukocytes recruited to an inflammatory site, with critical roles in phagocytosis and recruitment of other immune cells [73]. The release of NETs from neutrophils is crucial to immune defence and autoimmunity. However, exacerbated NETs foster chronic inflammation and controlling NETs has become a relevant therapeutical target for various non-infectious and autoimmune diseases [27, 32].

We herein show for the first time that NETs are significantly elevated in the blood of early schizophrenia patients relative to both their unaffected siblings and healthy controls. Increased levels of NETs were irrespective of sex, BMI, tobacco smoking, and other recreational drugs. These results extend previous findings of elevated neutrophils in schizophrenia [15–20], by mechanistically showing that these cells are in an active inflammatory state. It is becoming increasingly apparent that the release of NETs from neutrophils upon activation has a central role in both the initiation and chronicity of autoimmune and inflammatory diseases [32]. Some of the NETs-associated diseases, such as psoriasis, diabetes, and cardio-metabolic disorders, are comorbidities for psychosis and schizophrenia that contribute to a substantial reduction in life expectancy [25–32]. Therefore, targeting neutrophil function may help controlling immune dysfunction to improve quality of life and life expectancy in schizophrenia.

Whether increased NETs found in the blood of patients could affect the central nervous system (CNS) is unknown. However, a recent study in mice showed that inhibition of NETs using a genetic approach (knockout for PAD4, an enzyme that induces NETs formation) prevented lipopolysaccharide-induced depressive-like behaviour, besides peripheral (IL-6, TNF-α) and CNS inflammation (TNF-α, IL1-β) [74]. Another study showed that repeated social defeat in mice increased NETs formation from the bone marrow, which could have contributed to depressive-like behaviours and aortic atherosclerosis since both abnormalities were prevented by NETs inhibitor DNase I [75]. Congruently, a recent study by Vaibhav et al. (2020) showed that peripheral NETs formation in patients with traumatic brain injury was associated with worse neurological function [76]. Using mice, the authors showed that experimental traumatic brain injury induced impaired memory recognition and central NETs formation via activation of toll-like receptor 4, both of which were prevented by NETs inhibitor DNase I. These experimental studies indicate that the formation of peripheral NETs has a central effect and therefore investigating NETs in schizophrenia could help uncover novel mechanisms and treatment targets.

Unlike our expectations, however, increased NETs were not accompanied by elevated IL-6 in the overall patient sample. We focused on IL-6 in the current investigation given consistent evidence on the association between IL-6 and psychosis [10, 12, 77]. In our previous study, however, which included a larger but more heterogenous sample (first episode of affective and non-affective psychosis patients), IL-6 levels were significantly elevated in patients compared to both community controls and unaffected siblings [40]. The present non-significant results could indicate the effect of illness stage on IL-6 levels. In a meta-analysis by Pillinger et al. (2018), mean levels of IL-6 were significantly elevated in drug-naïve FEP compared to controls [10]. The meta-analysis by Miller et al. (2011), however, suggested that IL-6 levels may vary according to clinical status. IL-6 levels were increased relative to controls in both drug-naïve FEP and acutely relapsed inpatients but not in stable medicated outpatients [78]. In addition, IL-6 levels significantly normalised in FEP and acutely relapsed inpatients after antipsychotic treatment. Although we found no correlation between IL-6 levels and duration of pharmacological treatment, our patients were relatively stable at the time of assessment, which could be an explanation for the lack of significant results. Alternatively, it could suggest that elevated IL-6 levels are more pronounced only in a subgroup of patients, including those exposed to early stressful events.

### Antipsychotics do not increase NETs

Elevated NETs in our patients were not correlated with the duration of pharmacological treatment. This result was further supported by our in vitro assay using fresh neutrophils from a healthy donor; neither haloperidol nor risperidone induced NETs release. Instead, we observed the potential of first- and second-generation antipsychotics in inhibiting neutrophil-produced NETs induced by the stimulant PMA. Accordingly, meta-analyses show that increased neutrophil-to-lymphocyte ratio (NLR) observed in non-affective psychosis is unrelated to drug treatment [79], and that a significant decrease in neutrophil counts and NLR is observed after antipsychotic initiation [16]. Together, these results suggest that elevated neutrophils and related NETs in early schizophrenia are unlikely the result of pharmacological treatment.

In addition, elevated NETs in our patients were not correlated with clinical variables such as duration of psychosis, psychosis age of onset, DUP, or BPRS. In two previous studies, increased blood neutrophils were associated with the severity of symptoms in FEP patients [16, 80]. Others showed high neutrophils correlated with decreased grey matter volume and enlarged ventricles in FEP patients [81]. However, in a recent two-year longitudinal study in FEP, increased NLR did not correlate with the Positive and Negative Symptoms Scale scores but predicted poor remission at follow-up [20]. A previous study found similar results, such that NLR decreased following treatment only in the responsive group [82]. The lack of correlations in our study, especially with the severity of symptoms, could be explained by the fact that our patients were relatively stable (BPRS mean [SD] = 8.4 [6.2]). Alternatively, the apparent heterogeneous results could reflect the distinct measurements of neutrophils (absolute counts, ratio, or function). Unfortunately, in our study, absolute numbers or ratios were not available. Our focus, however, was on measures of cell function (NETs), a novel mechanism that can be applied to future larger longitudinal samples.

### A history of childhood maltreatment identifies subgroups of patients with higher NETs and IL-6 levels

Schizophrenia pathophysiology is likely the result of complex biological-environment associations [83–85]. Having identified higher NETs in patients compared to controls, our next step was to test potential environmental triggers associated with the immunological profile. Meta-analyses show that childhood maltreatment is associated with an elevated risk for mental and physical diseases [38, 39]. Associations between childhood maltreatment and inflammatory molecules in schizophrenia exist but are small and conflicting. For instance, associations between IL-6 and a history of childhood maltreatment have only been investigated in four studies among patients with schizophrenia or psychosis [86]. Hence, it is still an open debate whether childhood maltreatment could help subset patients with low-grade inflammation.

Previous studies using unsupervised two-step clustering analyses suggested that only a subset of patients with psychosis has signs of inflammation [41–46]. However, these studies did not consider environmental factors in their analyses. Only one study by Tamouza et al. (2021) included childhood maltreatment in clustering analyses [70]; however, their patient sample size was smaller (*n* = 18) and included chronically treated patients with schizophrenia. We applied the same method but in a much larger sample of early schizophrenia to avoid confounding by the long duration of treatment and illness on the measured biological outcomes. In our study, a history of childhood maltreatment was the most important variable contributing to cluster separation.

We also found that our identified clusters significantly differed on biological variables. Independent of diagnosis, 38.7% of the participants who reported a higher history of childhood maltreatment had concomitantly higher levels of NETs and IL-6 compared to participants allocated in the lower early-stressed cluster (61.3%), independent of covariates. The results suggest that considering a history of childhood maltreatment in future clustering analyses could help to subset distinct immune-inflammatory profiles and identify potential environmental triggers.

When considering diagnostic groups, most patients were allocated to the high early-stressed cluster (61.5%), while the opposite was found for both their unaffected siblings and controls. Interestingly, levels of NETs were higher in patients with high early stress compared to all the remaining groups (patients with lower early stress and siblings and controls with high or lower early stress). However, among the lower early-stressed clusters, patients still had higher NETs than controls. These results indicate that having a diagnosis of schizophrenia itself impacts NETs levels but adding a history of childhood trauma greatly elevates NETs levels in patients. Our exploratory analyses suggested that forms of neglect (emotional and physical) together with emotional abuse were the subtypes mostly contributing to cluster separation. This is consistent with evidence showing that the prevalence of child neglect is around 78% in the general population, while physical and sexual abuse are reported in around 18% and 9% [87].

For IL-6 levels, however, participants (patients, siblings, and controls) allocated in the high early-stressed cluster did not differ from each other. Instead, a specific effect was found between patients in the high early-stressed versus participants (patients, siblings, and controls) that pertained to the low early-stressed group. Our IL-6 results are similar to those found in the Tamouza study, in which IL-6 was high only in the chronic schizophrenia patient group reporting childhood maltreatment [70]. However, different from NETs, we did not find an effect of specific maltreatment subtypes on IL-6 results. This may suggest that a combination of stressful subtypes (based on total scores) is related to IL-6 changes in our sample. Together, these results align with a hypothesis that IL-6 is elevated only in a subset of patients, with childhood trauma being a potential trigger.

### Adolescent stress in rodents increases levels of NETs and IL-6

Further supporting our clinical findings and the role of psychological stress in immune dysregulation, we experimentally demonstrated that rats exposed to stress during adolescence had increased levels of NETs and IL-6 in serum compared to non-stressed rats. Additionally, fresh neutrophils isolated from the bone marrow of stressed rats released more NETs.

Recent findings from our group indicate that a combined protocol of stress applied during early adolescence leads to neural circuitry and behavioural deficits related to schizophrenia in late adolescence/adulthood. For example, adolescent stressed rats show increased anxiety-like behaviours, decreased sociability, cognitive deficits, and increased responsivity to psychostimulants, along with hippocampal hyperactivity, parvalbumin neuron loss, and an overactive dopamine system [66, 67]. Such changes are similar to those found in other animal models relevant to schizophrenia [88, 89] and consistent with clinical schizophrenia [90].

Stress, including psychological stress, can increase haematopoiesis and affect the number of neutrophils in circulation via stimulation of the sympathetic nervous system [91]. Thus, it is tempting to speculate that the increased NETs related to early stress observed in our study result from cell proliferation in the bone marrow and, consequently, their release in circulation. These events, along with NETs production, are likely to contribute to the release of inflammatory cytokines, such as IL-6, which together could contribute to stress-related neurobiological changes seen in schizophrenia.

### Strengthens, limitations and future directions

Our study has several strengths. This is the first study evaluating neutrophil function in early schizophrenia, bringing a novel cellular mechanism into the field. Second, different from previous studies, we integrated biological and environmental variables to test their combined contribution to cluster separation. Our sample included patients in their early stages of the disease to limit the effect of long duration of illness and chronic treatment. Finally, we included an in vitro assay to discard the effect of antipsychotic drugs on NETs formation and complemented our study with a rodent model to further test our hypothesis. Nonetheless, important limitations should be addressed. We do not have information on neutrophil counts or NRL in the blood of our clinical sample. However, NETs are a functional marker of neutrophils, and our results provide an important mechanistic extension to early findings of elevated neutrophils in schizophrenia. Our sibling sample size was small, limiting our investigation on the role of familial liability. Nonetheless, our patient and control sample sizes were much larger than a previous study conducted in chronic schizophrenia [70]. Future studies should attempt to measure neutrophils, NLR, and NETs and their longitudinal association with environmental factors, response to treatment, and symptom dimensions.

## Conclusions

Our study provides new mechanistic support to the involvement of neutrophils in schizophrenia by showing for the first time increased functional NETs in the blood of patients. We further suggest that NETs and early stress should be considered in future studies attempting to identify immune biological subgroups in schizophrenia. This study offers potential for further experimental medicine studies targeting NETs inhibitors.

## Supplementary information

SUPPLEMENTAL MATERIAL
