# Supplementary material for: Increased blood neutrophil extracellular traps (NETs) associated with early life stress: translational findings in recent-onset schizophrenia and rodent model
Source: Transl Psychiatry. 2022 Dec 26;12:526. doi: 10.1038/s41398-022-02291-4 (PMC9792518; doi:10.1038/s41398-022-02291-4)
Supplement: Supplementary file 1 — SUPPLEMENTAL MATERIAL [file 41398_2022_2291_MOESM1_ESM.docx]

**Supplementary material**

**Increased blood Neutrophil Extracellular Traps (NETs) associated with**

**early life stress: translational findings in recent-onset schizophrenia and rodent model**

Fabiana Corsi-Zuelli, MSc, Ayda Henriques Schneider, MSc, Thamyris Santos-Silva, MSc, Camila Marcelino Loureiro, PhD, Rosana Shuhama, PhD, Paulo Rossi Menezes, MD, PhD, Francisco Silveira Guimarães, MD, PhD, Felipe Villela Gomes, PhD, Fernando Cunha, PhD, Paulo Louzada-Junior, MD, PhD, Cristina Marta Del-Ben, MD, PhD

**Materials and Methods**

**Participants**

The current investigation is a subsample of the cross-sectional study and included all the recruited patients with early schizophrenia spectrum (n=78), sex- and age-matched controls (n=78), and available unaffected siblings of patients with early schizophrenia spectrum (n=25). If power was to be predetermined, our sample would provide a large effect size (Cohen's F = 0.5), assuming alpha=0.05, 80% of power, two-side significant threshold of 5%, considering a case-sibling-control study and the difference of mean and standard deviation of NETs between groups from our previous study (Schneider et al., 2021). For f-tests (ANOVA), Cohen suggests that F values of 0.1, 0.25, and 0.4 represent small, medium, and large effect sizes, respectively (calculated using RStudio Team (2020)).

**Biological samples**

*Quantification of NETs in human plasma*

An antibody bound to a 96-well clear-bottom black plate captured the enzyme myeloperoxidase (MPO) (Thermo Fisher Scientific, USA; CAT: PA5-16672), and the amount of DNA bound to the enzyme was quantified using the Quant-iT PicoGreen kit (Thermo Fisher Scientific, USA; CAT: P11496), according to the manufacturer’s instructions. The fluorescence intensity (excitation at 488 nm and emission at 525 nm wavelength) was determined by a FlexStation 3 microplate reader (Molecular Devices, USA).

**Rodent model**

Adolescent male rats were exposed to a combination of daily inescapable footshock (FS; from PD31-40) and three restraint stress (RS) sessions (PD31, 32, and 40). Briefly, rats were exposed to one session of FS per day for ten consecutive days. In each session, 25 scrambled FS (1.0 mA, 2 seconds) were delivered pseudo-randomly (5 cycles of 30, 60, 40, 60, and 90 seconds). On the first, second, and last day, immediately after the FS exposure, rats were submitted to RS in a Plexiglas cylindrical size-adjusted restraint tube. Each RS session lasted 1h, and immediately after the end of the RS, rats were returned to their home cages.

**Statistical analysis**

*NETs, IL-6, and childhood maltreatment; subgroups identification*

We used unsupervised two-step clustering analyses [43, 69, 70] to identify potential subgroups and account for heterogeneity in measures of NETs, IL-6, and childhood maltreatment. Two-step clustering was chosen to avoid bias when pre-determining cluster numbers after integrating variables of interest. The two steps included, firstly, a pre-clustering step that scanned the entire dataset and stored dense regions of data records in summary statistics. This was then followed by a hierarchical clustering algorithm. The Bayesian Criterion (BIC) estimated the maximum number of clusters, and the log-likelihood method determined the distance measured to separate groups.

**References**

Schneider AH, MacHado CC, Veras FP, Maganin AGDME, De Souza FFL, Barroso LC, et al. Neutrophil extracellular traps mediate joint hyperalgesia induced by immune inflammation. Rheumatology (Oxford). 2021;60:3461–3473.

R Core Team (2020). R: A language and environment for statistical computing. R Foundation for Statistical Computing, Vienna, Austria. URL https://www.R-project.org/.

**Results**

**Clinical sample**

*NETs are elevated in early schizophrenia patients*

| **Supplementary Table 1: Group difference in NETs and IL-6 plasma levels** | | | | | | |
| --- | --- | --- | --- | --- | --- | --- |
| **Model** | **Group difference** | | | | | |
|  | **Controls**  **(n= 78)** | **Patients**  **(n=78)** | **Siblings**  **(n=25)** | **Statistics** | | |
|  | Mean (SD) or estimated means (SEM) | | | F | df | p |
| **NETs** |  | | | | | |
| Unadjusted | 1.27 (0.64) | 3.31 (1.67) | 1.68 (1.39) | 76.37 | 2 | **<0.001^a,c^** |
| Adjusted^*^ | 1.55 (0.18) | 3.35 (0.16) | 1.89 (0.27) | 50.79 | 2 | **<0.001^a,c^** |
| **IL-6** |  | | | | | |
| Unadjusted | 1.82 (0.91) | 2.23 (1.70) | 1.79 (0.95) | 2.407 | 2 | 0.093 |
| Adjusted^*^ | 1.80 (0.19) | 2.2.4 (1.62) | 1.67 (0.28) | 2.704 | 2 | 0.070 |

Unadjusted models were performed using Univariance Analyses of Variance (ANOVA); mean, and SD are shown.

^*^Adjusted models were performed using Univariate Analysis of Covariance (ANCOVA) adjusted for sex, tobacco smoking, psychoactive substance use, and body mass index. For adjusted analyses, estimated means and SEM are represented.

Pairwise comparison: ^a^ Patients *v.* Siblings; ^b^ Siblings *v.* Controls; ^c^ Patients *v.* Controls.

For both unadjusted and adjusted models, the raw values are represented, while statistics were computed using the natural log-transformed values.

**Significant results are depicted in bold.**

**Results**

**Clinical sample**

*Plasma NETs, IL-6, and childhood maltreatment; subgroups identification*

**

**Supplementary Figure 1:** We used a two-step clustering analysis [15–17] to pre-determine cluster numbers after integrating variables of interest (CTQ total scores, NETs and IL-6 levels) in the whole sample (n=181). The two steps included firstly a pre-clustering step that scanned the entire dataset and stored dense regions of data records in summary statistics. This was then followed by a hierarchical clustering algorithm. The Bayesian Criterion (BIC) estimated the maximum number of clusters, and the log-likelihood method determined the distance measured to separate groups. CTQ total scores were the most important variable contributing to cluster separation, followed by NETs levels. IL-6 levels were the least contributor The cluster analysis identified two main clusters (CL1 and CL2) with good cluster separation (silhouette measure of cohesion and separation > 0.5).

CTQ: Childhood Trauma Questionnaire; NETs: Neutrophil Extracellular Traps; IL (Interleukin)-6.

**Results**

**Clinical sample**

*NETs, IL-6, and childhood maltreatment; subgroups identification*

**Supplementary Table 2: NETs and IL-6 plasma levels among clusters (high-CL1 and low-CL2)**

| **Model** | **Group difference** | | | | |
| --- | --- | --- | --- | --- | --- |
|  | **High-CL1**  **(n=70)** | **Low-CL2**  **(n=111)** | **Statistics** | | |
|  | Mean (SD) or estimated means (SEM) | | F | df | p |
| **NETs** |  | | | | |
| Unadjusted | 3.31 (1.95) | 1.51 (0.74) | 80.57 | 1 | **<0.001** |
| Adjusted^*^ | 3.34 (1.16) | 1.80 (1.15) | 56.66 | 1 | **<0.001** |
| **IL-6** |  | | | | |
| Unadjusted | 2.48 (1.88) | 1.68 (0.65) | 17.31 | 1 | **<0.001** |
| Adjusted^*^ | 2.44 (0.15) | 1.52 (0.15) | 19.59 | 1 | **<0.001** |

The sample (n=181) was stratified into cluster 1 (high-CL1) or cluster 2 (Low-CL2) using unsupervised two-step cluster analysis. Two main clusters were identified when integrating childhood maltreatment scores, NETs, and IL-6 levels. Cluster 1 (high-CL1) represents participants with higher scores of childhood maltreatment and levels of NETs and IL-6, whereas cluster 2 (low-CL2) represents the opposite.

Unadjusted models were performed using Univariance Analyses of Variance (ANOVA); mean and SD are shown.

^*^Adjusted models were performed using Univariate Analysis of Covariance (ANCOVA) adjusted for tobacco smoking and other psychoactive substance use. For adjusted analyses, estimated means and SEM are represented.

For both unadjusted and adjusted models, the raw values are represented, while statistics were computed using the natural log-transformed values.

**Significant results are depicted in bold**.

**Results**

**Clinical sample**

*Plasma NETs, IL-6, and childhood maltreatment; subgroups identification*

**Supplementary Table 3: Childhood Trauma Questionnaire (CTQ) total score among clusters (high-CL1 and low-CL2) in community controls, patients with early schizophrenia and their unaffected siblings**

| **Model** | **Group difference** | | | | | | | | |
| --- | --- | --- | --- | --- | --- | --- | --- | --- | --- |
|  | **High-CL1**  **(n=14)** | **Low-CL2**  **(n=64)** | **High-CL1**  **(n=48)** | **Low-CL2**  **(n=30)** | **High-CL1**  **(n=8)** | **Low-CL2**  **(n=17)** | **Statistics** | | |
| **CTQ total scores** | **Controls** | | **Patients** | | **Siblings** | |  |  |  |
|  | Mean (SD) | | | | | | F | df | p |
|  | 50.43 (16.02) | 31.27 (5.77) | 46.42 (15.34) | 31.43 (3.70) | 58.50 (10.14) | 33.12 (5.20) | 26.23 | 5 | **<0.001**  ^a,b,c,d^ |

High (CL1) and low (CL2) clusters were identified using unsupervised two-step cluster analysis after integrating childhood maltreatment scores, NETs, and IL-6 levels.

Unadjusted models were performed using Univariance Analyses of Variance (ANOVA); mean and SD are shown.

Pairwise comparison: ^a^ Patients high-CL1 *v.* Patients, siblings, and controls low-CL2; ^b^ Patients low-CL2 *v.* controls and siblings high-CL1; ^c^ Siblings high-CL1 *v.* patients high-CL1 and siblings and controls low-CL2; ^d^ Controls high-CL1 *v.* controls and siblings low-CL2.

**Significant results are depicted in bold**.

**Clinical sample**

*Plasma NETs, IL-6, and childhood maltreatment; subgroups identification*

**Supplementary Table 4: NETs and IL-6 levels among clusters (high-CL1 and low-CL2) in community controls, early schizophrenia patients and their unaffected siblings**

| **Model** | **Group differences** | | | | | | | | |
| --- | --- | --- | --- | --- | --- | --- | --- | --- | --- |
|  | **High-CL1**  **(n=14)** | **Low-CL2**  **(n=64)** | **High-CL1**  **(n=48)** | **Low-CL2**  **(n=30)** | **High-CL1**  **(n=8)** | **Low-CL2**  **(n=17)** | **Statistics** | | |
|  | **Controls** | | **Patients** | | **Siblings** | |  |  |  |
|  | Estimated means (SEM) | | | | | | F | df | p |
| **NETs** |  |  |  |  |  |  |  |  |  |
| Adjusted^*^ | 1.88 (0.32) | 1.30 (0.18) | 3.98 (0.17) | 2.27 (0.22) | 2.06 (0.41) | 1.71 (0.30) | 25.17 | 5 | **<0.001^a,b^** |
| **IL-6** |  |  |  |  |  |  |  |  |  |
| Adjusted^*^ | 2.15 (0.36) | 1.56 (0.21) | 2.59 (0.19) | 1.63 (0.25) | 2.15 (0.46) | 1.37 (0.34) | 3.87 | 5 | **0.002^c^** |

High (CL1) and low (CL2) clusters were identified using unsupervised two-step cluster analysis after integrating childhood maltreatment scores, NETs, and IL-6 levels.

^*^Adjusted models were performed using Univariate Analysis of Covariance (ANCOVA) adjusted for sex, tobacco smoking, and psychoactive substance use.

For adjusted analyses, estimated means and SEM are represented.

Pairwise comparison (NETs): ^a^ Patients high-CL1 *v.* Patients Low-CL2 and siblings and controls high-CL1 and low-CL1; ^b^ Patients low-CL2 *v.* controls low-CL2.

Pairwise comparison (IL-6): ^c^ Patients high-CL1 v. patients, siblings, and controls low-CL2.

**Significant results are depicted in bold.**

**Results**

**Clinical sample**

*Contribution of childhood maltreatment subtypes to cluster separation*

Mean scores and frequency of childhood maltreatment subtypes are detailed in **Supplementary Table 5.**

**Supplementary Table 5. Subtypes of childhood maltreatment (n=181)**

| **CTQ subtypes** | **Controls** | **Patients** | **Siblings** | **p** |
| --- | --- | --- | --- | --- |
|  | **(n= 78)** | **(n=78)** | **(n=25)** |  |
| Scores, mean (SD)**^1^** |  |  |  |  |
| Emotional neglect | 9.18 (5.25) | 10.55 (5.40) | 11.12 (5.94) | 0.065 |
| Physical neglect | 6.53 (2.88) | 7.41 (3.18) | 7.36 (2.87) | **0.013 ^a^** |
| Emotional abuse | 7.60 (3.56) | 9.28 (4.60) | 9.60 (3.63) | **0.003 ^a,b^** |
| Physical abuse | 6.32 (2.58) | 7.41 (3.67) | 7.64 (4.17) | 0.134 |
| Sexual abuse | 5.09 (0.56) | 6.00 (3.13) | 5.52 (1.50) | **0.032 ^a^** |
|  | | | | |
| Frequency, n (%) |  |  |  |  |
| Emotional neglect ^2^ | 14 (17.9) | 19 (24.4) | 6 (24.0) | 0.591 |
| Physical neglect ^3^ | 11 (14.1) | 17 (21.8) | 5 (20.0) | 0.480 |
| Emotional abuse ^2^ | 12 (15.4) | 16 (20.5) | 6 (24.0) | 0.552 |
| Physical abuse ^3^ | 5 (6.4) | 17 (21.8) | 5 (20.0) | **0.016 ª^,b^** |
| Sexual abuse ^3^ | 7 (9.0) | 2 (2.6) | 3 (12.0) | 0.098 |

^1^ Kruskal-Wallis; ^2^ Pearson Chi-square Test; ^3^ Fisher’s Exact Test.

Pairwise comparison: ^a^ Patients *v.* Controls; ^b^ Siblings *v.* Controls; ^c^ Patients *v.* Siblings.

CTQ: Childhood maltreatment questionnaire.

**Significant results are depicted in bold.**

Our exploratory analysis testing the impact of subtypes of childhood maltreatment on cluster separation showed that emotional neglect, physical neglect, and emotional abuse had the strongest impact. Physical and sexual abuse were the least contributors **(Supplementary Figure 2).**

**

**Supplementary Figure 2:** CTQ emotional neglect (EN), physical neglect (PN), and emotional abuse (EA) scores were the most important variable contributing to cluster separation. CTQ scores of physical abuse (PA) or sexual abuse (SA) were the least contributors. Silhouette measure of cohesion and separation > 0.5, n=181. CTQ: Childhood Trauma Questionnaire.

Unsupervised two-step clustering analysis integrating values of childhood maltreatment subtypes (emotional, physical and sexual abuse; emotional and physical neglect), NETs, and IL-6 in the whole sample (n=181) identified two main clusters (CL1_subtype and CL2_subtype). 22.7% (41 out of 181) participants were identified as CL1_subtype, while the remaining 77.3% (140 out of 181) were allocated to CL2_subtype.

When testing differences in sociodemographic variables, we observed that clusters did not differ in age or body mass index (p>0.05). However, CL1_subtype had more females (CL1_ subtype: 53.7% vs CL2_ subtype: 30.7%, p=0.007), higher frequency of tobacco smoking (CL1_subtype: 41.5% vs. CL2_subtype: 18.6%, p=0.002), and psychoactive substance misuse (CL1_ subtype: 56.1% vs. CL2_subtype: 33.6%, p=0.009) than CL2_subtype. In addition, CL1_subtype had significantly higher scores of childhood maltreatment subtypes than CL2_subtype (all p<0.001), see **Supplementary Table 6.**

**Supplementary Table 6: Scores of childhood maltreatment subtypes among clusters (high-CL1_subtype and low-CL2_subtype) in community controls, patients with early schizophrenia and their unaffected siblings**

| **Childhood maltreatment subtypes Mean (SD)** | **High-CL1_subtype** | **Low-CL2_subtype** | **p^1^** |
| --- | --- | --- | --- |
|  | **(n=41)** | **(n=140)** |  |
| Emotional neglect | 17.34 (4.99) | 7.90 (3.28) | **<0.001** |
| Physical neglect | 11.02 (3.64) | 4.85 (1.40) | **<0.001** |
| Emotional abuse | 13.68 (4.23) | 7.11 (2.65) | **<0.001** |
| Physical abuse | 10 (5.38) | 6.09 (1.66) | **<0.001** |
| Sexual abuse | 6.85 (4.13) | 5.16 (0.81) | **<0.001** |

High (CL1_subtype) and low (CL2_subtype) clusters were identified using unsupervised two-step cluster analysis after integrating scores of childhood maltreatment subtypes, NETs, and IL-6 levels.

^1^ Kruskal-Wallis test. **Significant results are depicted in bold**.

In addition, CL1_subtype had significantly higher NETs than CL2_subtype (p<0.001), in unadjusted analysis **(Supplementary Table 7).**

**Supplementary Table 7: NETs and IL-6 plasma levels among clusters by childhood maltreatment subtypes (high-CL1_subtype and low-CL2_subtype)**

| **Model** | **Group difference** | | | | |
| --- | --- | --- | --- | --- | --- |
|  | **High-CL1_subtype**  **(n=41)** | **Low-CL2_subtype**  **(n=140)** | **Statistics** | | |
|  | Mean (SD) or estimated means (SEM) | | F | df | p |
| **NETs** |  | | | | |
| Unadjusted | 2.75 (1.96) | 2.05 (1.45) | 6.474 | 1 | **0.012** |
| Adjusted^*^ | 2.77 (0.23) | 2.45 (0.16) | 1.438 | 1 | 0.232 |
| **IL-6** |  | | | | |
| Unadjusted | 2.08 (1.24) | 1.97 (1.35) | 0.396 | 1 | 0.530 |
| Adjusted^*^ | 2.04 (0.21) | 2.01 (0.15) | 0.099 | 1 | 0.754 |

The sample (n=181) was stratified into cluster 1 (high-CL1_subtype) or cluster 2 (Low-CL2_subtype) using unsupervised two-step cluster analysis. Two main clusters were identified when integrating values of childhood maltreatment subtypes (emotional, physical and sexual abuse; emotional and physical neglect), NETs, and IL-6 levels. Cluster 1 (high-CL1_subtype) represents participants with higher scores of childhood maltreatment subtypes, mean levels of NETs and IL-6, whereas cluster 2 (low-CL2_subtype) represents the opposite.

Unadjusted models were performed using Univariance Analyses of Variance (ANOVA); mean and SD are shown.

^*^Adjusted models were performed using Univariate Analysis of Covariance (ANCOVA) adjusted for sex, tobacco smoking and other psychoactive substance use. For adjusted analyses, estimated means and SEM are represented.

For both unadjusted and adjusted models, the raw values are represented, while statistics were computed using the natural log-transformed values.

**Significant results are depicted in bold**.

As a next step, we used the preceding cluster separation of childhood trauma subtypes to perform the same analyses but now considering diagnoses for group stratification.

Subgroup differences for IL-6 and NETs values are detailed on **Supplementary Table 8.** Briefly, patients high-CL1_subtype and low-CL2_subtype had higher NETs than the remaining groups (p<0.001, adjusted analysis).

**Supplementary Table 8: NETs and IL-6 levels among clusters (high-CL1_subtype and low-CL2_subtype) in community controls, early schizophrenia patients and their unaffected siblings**

| **Model** | **Group differences** | | | | | | | | | |
| --- | --- | --- | --- | --- | --- | --- | --- | --- | --- | --- |
|  | **High-CL1**  **(n=12)** | **Low-CL2**  **(n=66)** | **High-CL1**  **(n=22)** | | **Low-CL2**  **(n=56)** | **High-CL1**  **(n=7)** | **Low-CL2**  **(n=18)** | **Statistics** | | |
|  | **Controls** | | **Patients** | | | **Siblings** | |  |  |  |
|  | Estimated means (SEM) | | | | | | | F | df | p |
| **NETs** |  |  |  |  | |  |  |  |  |  |
| Unadjusted | 1.50 (0.39) | 1.23 (0.66) | 3.89 (2.06) | 3.08 (1.44) | | 1.27 (0.35) | 1.84 (1.60) | 32.63 | 5 | **<0.001^a,b^** |
| Adjusted^*^ | 1.78 (0.38) | 1.50 (0.20) | 3.83 (0.26) | 3.18 (0.18) | | 1.22 (0.48) | 2.15 (0.32) | 23.14 | 5 | **<0.001^a,b^** |
| **IL-6** |  |  |  |  | |  |  |  |  |  |
| Unadjusted | 1.95 (0.97) | 1.80 (0.90) | 2.29 (1.83) | 2.08 (1.37) | | 2.30 (1.36) | 1.60 (0.69) | 1.479 | 5 | 0.199 |
| Adjusted^*^ | 1.71 (0.40) | 1.76 (0.21) | 2.16 (0.29) | 2.29 (0.19) | | 2.22 (0.51) | 1.42 (0.34) | 1.633 | 5 | 0.154 |

High (CL1) and low (CL2) clusters were identified using unsupervised two-step cluster analysis after integrating childhood maltreatment scores, NETs, and IL-6 levels.

^*^Adjusted models were performed using Univariate Analysis of Covariance (ANCOVA) adjusted for sex, tobacco smoking, and psychoactive substance use.

For adjusted analyses, estimated means and SEM are represented.

Pairwise comparison: ^a^ Patients high-CL1_subtype *v.* siblings and controls high and low; ^b^ Patients low-CL2_subtype *v.* siblings and controls high and low.

**Significant results are depicted in bold.**
